# Seroprevalence and Molecular Detection of Bovine Anaplasmosis in Egypt

**DOI:** 10.3390/pathogens9010064

**Published:** 2020-01-16

**Authors:** Omid Parvizi, Hosny El-Adawy, Falk Melzer, Uwe Roesler, Heinrich Neubauer, Katja Mertens-Scholz

**Affiliations:** 1 Institute of Bacterial Infections and Zoonoses, Friedrich-Loeffler-Institut (Federal Research Institute for Animal Health), Naumburger Str. 96a, 07743 Jena, Germany; Hosny.ElAdawy@fli.de (H.E.-A.); Falk.Melzer@fli.de (F.M.); Heinrich.Neubauer@fli.de (H.N.); Katja.Mertens-Scholz@fli.de (K.M.-S.); 2 Faculty of Veterinary Medicine, Kafrelsheikh University, Kafr El-Sheikh 33516, Egypt; 3 Institute for Animal Hygiene and Environmental Health, Free University, Berlin, Robert-von Ostertag-Str. 7-13, 14163 Berlin, Germany; Uwe.Roesler@fu-berlin.de

**Keywords:** *Anaplasma marginale*, Bovine anaplasmosis, *Coxiella burnetii*, Egypt, prevalence, ELISA, real time PCR

## Abstract

Bovine anaplasmosis is a tick-borne disease with zoonotic potential, caused by the obligate intracellular bacterium *Anaplasma marginale*. The disease is distributed worldwide in tropical and subtropical regions. The economic losses from anaplasmosis in animals is of significant importance because it causes severe morbidity and mortality in cattle. Recovered animals may become persistent carriers. Epidemiological information on the actual status of bovine anaplasmosis in Egypt is scarce. Thus, this study aimed to determine anti-*Anaplasma* antibody and DNA in serum samples using ELISA and PCR, respectively. In total, 758 bovine sera were collected from cattle farms located in 24 Egyptian governorates in 2015 to 2016. Sera were analyzed with the commercially available ‘*Anaplasma* antibody competitive ELISA v2’ kit and ‘AmpliTest *Anaplasma*/*Ehrlichia* spp. real time TaqMan ^TM^ PCR. *Anaplasma* spp. antibodies were detected in 140 (18.5%) (CI: 15.8–21.4%) of the investigated sera by ELISA, and Anaplasma/Ehrlichia-DNA was detected in 40 (5.3%) (CI: 3.8–7.1%) of the positive sera by real time PCR. Co-detection of both *Anaplasma* spp. and *Coxiella burnetii*-specific antibodies was proven in 30 (4%) of the investigated sera. The results of this work confirm the significant prevalence of bovine anaplasmosis in Egypt. Raising awareness in decision makers of the public health, veterinarians and animal owners is required to reduce the spread of infection.

## 1. Introduction

Bovine anaplasmosis is caused by the obligate intracellular bacterium *Anaplasma marginale*, (Alphaproteobacteria: Rickettsiales: Anaplasmataceae) that was first described by Sir Arnold Theiler in 1910 as the causative agent of gall sickness in cattle [1]. Anaplasmosis is a tick-borne disease and bacteria replicate within the epithelial cells of the tick midgut [2,3]. It is endemic in tropical and subtropical areas worldwide. Anaplasmosis could be misdiagnosed with other tick-borne diseases caused by *Babesia* (*B*.) *bovis* and *B. bigemina*, which have a similar geographical distribution and cause anemia in cattle [4]. Besides transmission by ticks, these hemoprotozoa and *A. marginale* can also be transmitted mechanically by biting flies [5], needles [6], ear-tagging, castration and dehorning equipment [7,8], and parasites of migratory wild birds [9,10].

Other *Anaplasma* species that may cause bovine anaplasmosis are *A. centrale* causing only a mild disease, and *A. bovis* and *A. phagocytophilum* known as bovine ehrlichiosis and tick-borne fever, respectively [11]. They can infect cattle and cause a reduction of milk production. Bovine congenital transmission was reported for *A. phagocytophilum* [12], which has been recognized as a zoonotic agent [8,13]. The severity of symptoms depends on several host factors such as its immune status and possible coinfections by other pathogens [13]. Symptoms occur after a latency period i.e., progressive anemia due to multiplication of *A. marginale* or *A. centrale* within mature erythrocytes. Other symptoms are fever, inappetence, loss of coordination, breathlessness, reduced growth rate, abortions, and stillbirth. Compared to other pathogenic bacteria, there is no report proving the transmission of *Anaplasma* spp. to humans via animal products [14]. In humans, blood transfusion and organ transplantation have been recognized as modes of transmission for *A. phagocytophilum* [15,16,17].

*Anaplasma* spp. in general have long life persistence and are able to remain in populations for months or years, which has a significant influence on spreading and new outbreaks of anaplasmosis [8,18,19]. Control measures should include regular monitoring, timely treatment and countermeasures against the arthropod vectors [5], but the feasibility depends on various factors such as geographic location and implementation costs of regulatory measures e.g., use of vaccines or antibiotics [20]. Variations of vector competence and limitations of our knowledge on the tick immune responses hinder control efforts and especially our understanding of the arthropod–microbe interaction [21]. Despite the limited current knowledge, a tick vaccine is already under development [22].

Bovine anaplasmosis is an economically important disease that causes losses in the dairy and beef industries through reduced milk production, weight loss, abortion, icterus, and even death in some cases [23,24]. There exists no reports on the antibiotic resistance of these pathogens. Tetracyclines and imidocarb are recommended by the World Organisation for Animal Health (OIE) to reduce probable side effects of an attenuated *A. centrale* live vaccine [6]. Marcondes reported on successful oxytetracycline treatment [25].

The NCBI database holds only two complete whole genome sequences of *A. marginale* and four of *A. phagocytophilum* isolates. Diagnostic assays used in veterinary medicine to identify *A. marginale* and *A. centrale* showed that the competitive ELISA (cELISA) test is recommend for monitoring and screening of populations while PCR and Giemsa are recommended for staining for the examination of clinical cases [6].

The average number of cattle kept per year in Egypt between 2002 and 2014 was more than 4.6 million, highlighting the importance of dairy and meat production in this country. Bovine anaplasmosis in Egypt was mentioned first in the national report of 1966 [26]. Since then, the disease was detected in many governorates. In Egypt, several studies reported anaplasmosis caused by *A. marginale* in cattle, water buffaloes and camel [27,28,29,30,31,32]. Frequently used techniques in these reports were microscopy [30], competitive ELISA (cELISA) [33,34], immunofluorescent assay (IFA) [35,36], or molecular assays i.e., conventional PCR [27] or real-time PCR [37].

Epidemiological studies are useful for the monitoring and control of diseases, and subsequently, the reduction of costs. For bovine anaplasmosis, such studies were limited to some governorates, and a comprehensive study for the whole of Egypt is missing. The objective of this study was to update the epidemiological information about bovine anaplasmosis in Egypt through investigating the prevalence of anaplasmosis in cattle within 27 Egyptian governorates using cELISA and real time PCR, to predict risk factors and provide baseline data for an effective design of disease control.

## 2. Materials and Methods

### 2.1. Study Area and Sample Information

**Figure 1:**
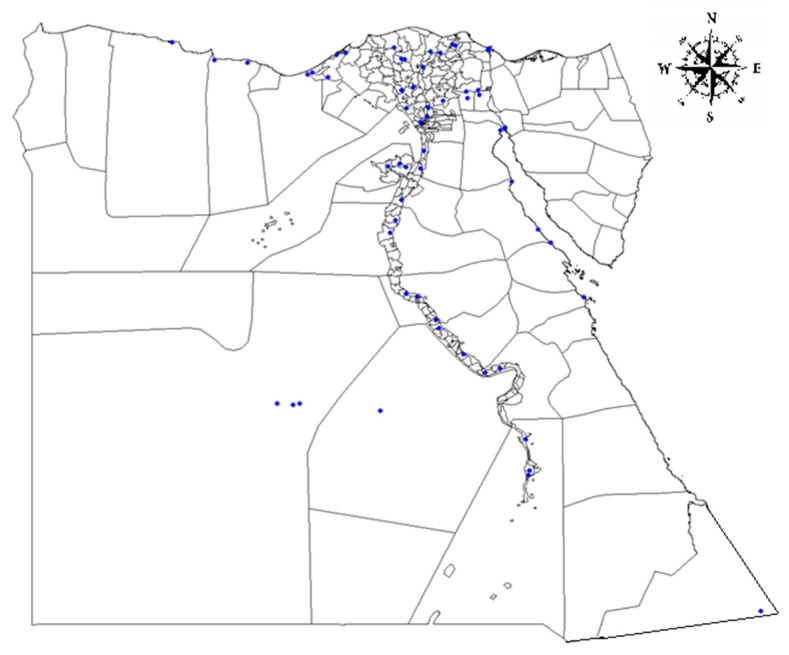
Sampling sites in Egypt. The map illustrates the position of sampling sites in each governorate.

Egypt is a vast desert plateau interrupted by the Nile valley and Delta region. Approximately 95% of the human population lives within 20 km of the Nile River and its delta. This territory is divided into 27 governorates, which have been categorized into three large domains: the Western part, the Eastern part and the Nile Valley and Delta region. In total, 758 cattle serum samples were collected during a Q fever prevalence study between October 2015 and March 2016 from 61 different farms located in 61 districts (sample sites) of 24 governorates (North Sinai, South Sinai and Luxor were excluded). A questionnaire that contained information about the animals, such as age, husbandry systems, infesting parasites, contact with other animals (i.e., dogs, etc.), and GPS data was used in this work (Figure 1). Age was categorized in two groups: ≤4 or >4 years. Three different husbandry systems were present: stable/stationary, pasture and nomadic.

The distribution of 758 cattle sera (Figure 1) was 283 (37.33%) from the Nile Delta domain, 337 (44.06%) from the Western domain, and 138 (18.2%) from the Eastern domain. Out of the 758 investigated cattle, 414 (54.61%) were kept in stables/stationary and 310 (40.89%) were nomadic. Tick infestation was recorded in 55.8% (n = 423), and 60.16% of animals were older than 4 years. All data regarding age group, animal housing and others are summarized in Table 1.

### 2.2. Detection of Anaplasma spp.-Specific Antibodies Using cELISA

Sera were stored at −20 °C and tested for specific antibodies against *Anaplasma* spp. using a competitive ELISA (cELISA) (Veterinary Medical Research and Development Inc., Pullman, WA, USA) according to the manufacturer’s instructions. The assay has a sensitivity of 100% and specificity of 99.7% according to the supplier [38]. All sera were tested in duplicate. Results were calculated according to manufacturer’s recommendation: percentage inhibition (% I) = 100 (1 − [sample OD620/OD620 of negative control]). Samples with a value ≥ 30% were considered as positive. 

### 2.3. Detection of Anaplasma spp./Ehrlichia spp. DNA Using Real Time PCR

The DNA was extracted and purified from all seropositive and suspected positive samples with only one positive cELISA result using High Pure PCR Template Preparation Kit (Roche, Mannheim, Germany) according to the manufacturer’s instructions. The concentration and quality of DNA was measured using a NanoDrop1000^®^ (Thermo Fisher, Wilmington, NC, USA) according to the manufacturer’s guidelines. The mean DNA concentration was 11.31 ± 6.9 ng/μL. DNA samples were stored at −20 °C until further use. For detection of *Anaplasma* spp./*Ehrlichia* spp.-specific DNA, the AmpliTest *Anaplasma* spp./*Ehrlichia* spp. Kit (Amplicon Ltd., Wrocław, Poland) was used according to the manufacturers guidelines. The assay has a sensitivity and specificity of 100% according to the manufacturer. The presence of *Anaplasma* spp. *Ehrlichia* spp.-specific DNA was determined in duplicates. A Ct value ≤ 38 was considered as positive and values between 38 and 40 were considered as uncertain results as recommended by the supplier. Species identification was performed on qPCR positive and questionable samples using conventional PCR targeting the 16S rRNA gene and sequencing as described elsewhere [39].

### 2.4. Statistical Analysis

Data analysis were performed with SPSS Statistics software^®^ (IBM Corp, Armonk, NY, USA, version 19). Seroprevalence is the proportion of positive results measured in serum within a population. Confidence interval (CI) was computed from binominal distribution of the obtained data (positivity in population). Odds ratios (ORs) were calculated using a relative risk option. In the present survey, possible risk factors such as age, tick infestation, animal husbandry system, age group (≤4 and >4 years), and keeping condition (stable/stationary, nomadic and posture) were analyzed. The Chi-square test was used to determine the association among categorized risk groups [40]. The multivariable regression model was used to evaluate the effect of multiple variables in the same model using ANOVA and F test for cELISA and real time PCR results.

### 2.5. Ethical Statement

This study was carried out in strict accordance with the recommendations of the Egyptian Network of Research Ethics Committees (ENREC), which complies with the international laws and regulations regarding ethical considerations in research. The ENREC approved this research work. For purposes of this study, all animal owners consented to sampling.

## 3. Results

Out of 758 tested serum samples, 140 were seropositive by cELISA, and the overall estimated seroprevalence of anaplasmosis was 18.5% (CI: 15.8–21.4%). In 61 investigated farms, 31 (50.8%) farms had seropositive cattle for anaplasmosis (Table 2). The results of seroprevalence for each governorates are shown in Table 2. The majority of seropositive animals were located in Gharbia (100%), Suez (83.3%) and Port Said (33.3%), while the lowest prevalence was recorded in Sohag (4.7%) and Aswan (5.2%). *Anaplasma*/*Ehrlichia*-specific DNA was detected in 5.3% (CI: 3.8–7.1%) of the seropositive samples by real time PCR. Species differentiation was attempted by 16S rRNA amplification and sequencing. Only four of all qPCR positive and questionable samples were positive for the 16S rRNA gene and showed 100% sequence identity with *A. marginale* (data not shown). Most of the PCR-positive animals were from the Nile Valley and Delta region (7.1%). Only 3.95% (30/758) of sera were serologically positive for *Coxiella burnetii* and *Anaplasma* spp. (Table 2).

The number of positive samples per domain ranged between 18.1–19.08% and 2.9–7.1%, respectively (Table 3).

Sixty percent of cELISA-positive animals were older than 4 years, with 56.42% of those animals kept in stables/stationary and 36.42% being nomadic. Sixty-five percent of positive animals were infested with ticks (Table 3). Tick infestation was the only risk factor that had a significant association with bovine anaplasmosis (χ^2^ = 9.36, *p* = 0.009), which is reflected by an Odds ratio of 1.7. Detailed information about this risk factor analyses is displayed in Table 3. The multivariable regression model demonstrated no relationship between risk factors of anaplasmosis (cELISA < ANOVA; F (6,744) = 0.799, *p* = 0.571> / real time PCR < ANOVA; F (6,744) = 2.005, *p* = 0.063>). 

## 4. Discussion

The aim of this study was to assess the prevalence of bovine anaplasmosis in Egypt to predict risk factors and provide baseline data for an effective design of disease control. Anaplasmosis has been recorded in cattle in Egypt for more than 50 years since it was first mentioned in 1966 [26], and was present in at least 22 of 27 governorates and the majority of positive samples reported from Suez, Dakahilia, Sharkir, Kafar el-Sheikh, Garbia, Manofia, and Minya [26]. Despite the evidence for endemicity of *Anaplasma* spp. in Egypt in official reports, a lack of data in the scientific literature is obvious. Only seven articles were found that provide data on anaplasmosis in cattle and one each in water buffaloes and camels. It is possible that the infections are more prevalent as reported, due to misdiagnosis and undetected carrier animals. It is not obvious why anaplasmosis does not get the expected attention from non-governmental scientists. This shows a strong need for more detailed information on the distribution of anaplasmosis in Egypt.

To understand the epidemiology of bovine anaplasmosis in Egypt, screening of sera collected for a previous Q fever survey were used to determine prevalence, risk factors and distribution of bovine anaplasmosis in Egypt.

In this study, the seroprevalence of anaplasmosis in Qena governorate was 50%, which is higher than that reported previously by Fereig et al. (28%) using a cELISA test [33]. Molecular investigation done by El-Ashkar et al. (2015) showed a high difference for the presence of *A. marginale*-specific DNA in sera when compared to the obtained data in this study, 20.12% vs 9.09%, respectively [27]. These discrepancies may be caused by different sampling times, sampling strategies and locations. It has to be noted that the samples in this study were taken on an independent, statistically-based sampling plan in contrast to sampling during locally limited outbreaks or samples taken from clinical practice. Most reports on bovine anaplasmosis were from animals, which were clinically ill or had a history of anaplasmosis. Screening by IFA was performed twice previously [35,36]. This test has several drawbacks i.e., limitations on the number of tests per day to be done by one operator and nonspecific fluorescence [6]. Hence, it is not recommended by OIE [6]. Studies using IFA for diagnosis of anaplasmosis cannot be compared to other studies using different OIE suitable assays i.e., cELISA. Six studies have used microscopic examination to confirm the agents near the margin of the erythrocyte. This method is recommended by the OIE for the confirmation of clinical cases of anaplasmosis. However, microscopy is not appropriate for prevalence studies and does not allow species differentiation [6]. The combination of cELISA and real time PCR proved to be easy in implementation in the laboratory and allows high throughput analysis of samples.

Chi square analyses resulted in a significant association for tick infestation with χ(2) = 9.36 and *p* = 0.009. This finding was expected, as ticks are vectors of anaplasmosis. There is no significant association between anaplasmosis and Q fever (χ(6) = 6.27, *p* = 0.18). In addition, the multivariable regression model indicated no dependency between risk factors and their relevance for anaplasmosis (cELISA < ANOVA; F (6,744) = 0.799, *p* = 0.571> / real time PCR < ANOVA; F (6,744) = 2.005, *p* = 0.063>). 

Summarized data from Egyptian literature [27,33,34,36], official reports, and this work show that bovine anaplasmosis is present in the governorates Matrouh, Damietta, Dakahila, and Qena (except Qalybia due to in-availability of samples). An inconsistency of national reports and our results for Sharkia and Beheira are based on limited availability of samples. No official reports from Aswan and Red sea were available, but in the presented study, 5.17% and 18.51% were positive by cELISA, and thereof, 3.44% and 2.89% were PCR-positive for *Anaplasma* spp./*Ehlichia* spp.-specific DNA, respectively. Species differentiation using conventional PCR targeting the 16S rRNA gene was not successful. Only four samples yielded PCR products with 100% sequence identity to *A. marginale*. This might be due to a higher sensitivity of the qPCR assay compared to conventional PCR and a low amount and quality of DNA. Beni-Suef is the only governorate in which no outbreaks have been reported and was also found to have no positive samples in this study. Bovine anaplasmosis is present in neighboring governorates, but why it is absent from this governorate is unknown. We found antibodies specific for bovine anaplasmosis in 17 governorates, which coincides well with official statistics. The country has an enormous burden of diseases and outbreaks; effective control of bovine anaplasmosis should include control of the tick vectors. In all domains, cattle were infested with ticks, which may be due to the unavailability of acaricides or access to information affecting the ability of animal owners to control ticks. This might also indicate that there is not sufficient veterinary care. Tick vaccines have a negative influence on tick feeding and reproduction [22] but are not available for field use yet. *Anaplasma centrale* live vaccines can give partial protection against bovine anaplasmosis and might be useful in future control programs. The role of nomadic husbandry in the dissemination of anaplasmosis is unknown and is still not investigated yet. The spread of diseases through human behavior [41], humans activities [42], and human mobility [43] are well known. The movement of carrier animals that do not display any obvious symptoms of anaplasmosis may be an additional factor to be considered.

## 5. Conclusions

National reports show that bovine anaplasmosis is widely distributed in Egypt. The results of this study confirm the nationwide and significant prevalence of bovine anaplasmosis. In order to reduce the spread of infection, more attention to control measures is required. Raising of awareness in decision makers of the public health and private sectors, especially veterinarians and animal owners, is an effective but simple way to improve the situation of anaplasmosis in a reasonably short time.

## Figures and Tables

**Table 1:** Number of animals sampled per domain with age group, husbandry system and tick infestation.

Domain		Western Domain	Nile Delta	Eastern Domain	Total
Cattle		334 (44.06%)	283 (37.33%)	135 (18.2%)	758
Animal age	≤4 years	175 (57.94%)	73 (24.17%)	54 (17.81%)	302 (39.84%)
	>4 years	162 (35.52%)	210 (46.05%)	84 (18.42%)	456 (60.16%)
Animal husbandry	Stable/Stationary	No samples	280 (67.63%)	134 (32.36%)	414 (54.61%)
	Nomadic	303 (97.74%)	3 (0.96%)	4 (1.29%)	310 (40.89%)
	Nomadic & Pasture	34	(-)	(-)	34 (4.48%)
Tick infestation		193 (45.62%)	149 (36.69%)	81 (19.14%)	423 (55.8%)
Cattle kept in spatial separate		(-)	280 (68.96%)	126 (31.03%)	406 (53.56%)
Others animal species living on farm		8 (32%)	15 (60%)	2 (8%)	25 (3.29%)

(-) No samples were available.

**Table 2:** Prevalence of bovine anaplasmosis in 24 investigated governorates.

Domain	Governorate	No. of Animals Tested	No. of Farms (Positive)	Prevalence No. (%)		Co-detection of *Coxiella* and *Anaplasma*
				cELISA	PCR	
Western Area	Matrouh	167	4 (4)	25 (15)	7 (4.2)	3
	New valley	170	6 (5)	36 (21.6)	9 (5.3)	8
Eastern Area	Red Sea	138	4 (3)	25 (18.5)	4 (2.9)	10
Nile Valley and Delta Area	Alexandria	9	3 (1)	1 (11.1)	0	0
	Assiut	33	2 (2)	10 (30.3)	2 (6.1)	2
	Aswan	58	3 (1)	3 (5.2)	2 (3.4)	2
	Cairo	12	2 (1)	2 (16.7)	0	0
	Dakahlia	11	2 (1)	2 (18.2)	1 (9.1)	0
	Damietta	12	2 (2)	3 (25)	3 (25)	0
	Fayoum	9	3 (2)	2 (22.2)	0	0
	Gharbia	2	1 (1)	2 (100)	2 (100)	0
	Ismailia	7	4 (2)	2 (28.5)	0	0
	Minya	12	2 (1)	1 (8.3)	1 (8.3)	0
	Port Said	12	2 (2)	4 (33.3)	3 (25)	0
	Qena	22	3 (2)	11 (50)	3 (13.6)	0
	Sohag	21	2 (1)	1 (4.8)	0	1
	Suez	12	2 (2)	10 (83.3)	3 (25)	4
	Beheira	1	1 (0)	0	0	0
	Beni-Suef	22	2 (0)	0	0	0
	Giza	9	3 (0)	0	0	0
	Kafr El Sheikh	7	3 (0)	0	0	0
	Menoufia	9	3 (0)	0	0	0
	Qualyubia	1	1 (0)	0	0	0
	Sharkia	2	1 (0)	0	0	0
Total		758	61 (33%)	140 (18.5%)	40 (5.3%)	30 (4%)

**Table 3:** Potential risk-associated factors for bovine anaplasmosis in Egypt.

		c ELISA							Real Time PCR			
Risk Factor		No. of Positive Animals (No. of Suspicious Samples)			Seropositive	Odds Ratio	95% Confidence Interval (CI) Pos. (Pos. plus Suspicious)	Chi Square (df) (*p*-Value)	No. of Positive Animals (Suspicious)	DNA Positive Samples	95% Confidence Interval (CI)	Chi Square (df) (*p*-Value)
			Proportion in Positive Animals (Suspicious)	Proportion in Total Animals (Suspicious)								
Domain	Western Domain	61 (22)	43.57% (55%)	61/337 = 18.10% (6.52%)	18.10%	1.09	14.1–22.6%	χ^2^(4) = 2.23; *p* = 0.69	16 (9)	4.74%	2.7–7.6%	χ^2^(6) = 9.01; *p* = 0.17
	Nile Delta	54 (11)	38.57% (27.5%)	54/283 = 19.08% (3.88%)	19.08%	0.92	14.7–24.2%		20 (1)	7.1%	4.4–10.7%	
	Eastern Domain	25 (7)	17.85 (17.5%)	25/138 = 18.11% (5.07%)	18.11%	0.99	12.1–25.6%		4 (3)	2.9%	0.8–7.3%	
	Total	140 (40)		140/758 = 18.46% (5.27%)	18.5%	ND	15.8–21.4%		40 (13)	5.3%	3.8–7.1%	
Animal age group	≤4 years	56 (17)	40% (42.5%)	18.54% (5.62%)	18.54%	1.02	14.3–23.4%	χ^2^(2) = 0.144; *p* = 0.93	19 (7)	6.3%	3.8–9.7%	χ^2^(3) = 2.57; *p* = 0.46
	>4 years	84 (23)	60% (57.5%)	18.42% (5.04%)	18.42%	0.98	15.0–22.3%		21 (6)	4.60%	2.9–7%	
Animal husbandry	Stable/Stationary	79 (18)	56.42% (45%)	19.08% (4.34%)	19.1%	0.96	15.4–23.2%	χ^2^(6) = 8.30; *p* = 0.21	24 (4)	5.8%	3.7–8.5%	χ^2^(9) = 8.82; *p* = 0.69
	Nomadic	51 (22)	36.42% (55%)	16.45% (7.09%)	16.5%	0.98	12.5–21.1%		13 (8)	4.2%	2.3–7.1%	
	Nomadic & Pasture	10	7.14%	29.41%	29.41%	1.34	15.1–47.5%		3 (1)	8.8%	1.9–23.7%	
Tick infestation		91 (27)	65% (67.5%)	21.51% (6.38%)	19.45%	1.71	17.7–25.7%	χ^2^(2) = 9.36; *p* = 0.009^a^	26 (11)	6.1%	4.1–8.9%	χ^2^(3) = 11.74; *p* = 0.45
Animals kept separate		79 (18)	56.42% (45%)	19.45% (4.43%)	19.5%	1.02	15.7–23.6%	χ^2^(2) = 1.64; *p* = 0.44	24 (4)	5.9%	3.8–8.7%	χ^2^(4) = 3.38; *p* = 0.33
Another animal species living on farm		6 (1)	25% (4%)	24% (4%)	24%	ND	9.4–45.1%	ND	1 (2)	4%	0.1–20.4%	ND

Chi-square analysis calculated by ignoring the missing samples to avoid a high percentage of expected frequency below 5.^a^ Demonstrated significant association for tick infestation. Both assays were conducted in duplicate. ‘suspicious’ means that samples have only one positive result.
